# Endovascular Gene Delivery from a Stent Platform: Gene- Eluting Stents

**DOI:** 10.4172/2329-9495.1000109

**Published:** 2013-07-27

**Authors:** Ilia Fishbein, Michael Chorny, Richard F Adamo, Scott P Forbes, Ricardo A Corrales, Ivan S Alferiev, Robert J Levy

**Affiliations:** 1 Dept of Pediatrics, Division of Cardiology, The Children’s Hospital of Philadelphia, USA; 2 The University of Pennsylvania, USA

**Keywords:** Endovascular stents, Restenosis, Vascular gene delivery

## Abstract

A synergistic impact of research in the fields of post-angioplasty restenosis, drug-eluting stents and vascular gene therapy over the past 15 years has shaped the concept of gene-eluting stents. Gene-eluting stents hold promise of overcoming some biological and technical problems inherent to drug-eluting stent technology. As the field of gene-eluting stents matures it becomes evident that all three main design modules of a gene-eluting stent: a therapeutic transgene, a vector and a delivery system are equally important for accomplishing sustained inhibition of neointimal formation in arteries treated with gene delivery stents. This review summarizes prior work on stent-based gene delivery and discusses the main optimization strategies required to move the field of gene-eluting stents to clinical translation.

## DES for the Prevention of In-Stent Restenosis

A percutaneous vascular intervention (PCI) is a standard therapeutic approach for treating patients with symptomatic coronary disease. PCI are carried out annually in more than one million patients in USA alone [1]. An overwhelming majority of PCI cases involve stent angioplasty performed with either bare metal stents (BMS) or drug-eluting stents (DES). Compared to BMS, DES achieve better outcomes related to early and mid-term arterial patency and are associated with a reduced need for target vessel revascularization [2]. However, in some studies DES have been linked to a higher rate of late stent thrombosis (LST) [3]. Aborted healing response, lack of re-endothelialization and unresolved inflammation have been suggested as the major factors contributing to this complication [3]. LST is a grave medical condition carrying a high risk of sudden cardiac death. To minimize the risk of thrombosis, patients implanted with DES are routinely prescribed prolonged aggressive dual anti-platelet therapy that causes bleeding complications in a significant fraction of post-PCI patients [4]. The incidence of ISR and LST varies widely among different groups of patients and lesion characteristics. Most registries based on unselected patient populations report 20–30% and 8–10% ISR, and 0.8–1% and 1.5–2% LST in the first year following stent deployment, for the patients receiving BMS and DES, respectively [5,6].

Moreover, the anti-restenotic effect of DES is less pronounced in patients with peripheral artery disease [7], as well as coronary disease patients afflicted by diabetes or renal failure [8]. Furthermore, the presence of ostial lesions, pre-existing ISR, smaller arterial diameter and longer lesion length are also associated with poor outcomes [8]. The binary rate of restenosis in patients with these and some other anatomical and clinical circumstances is currently in double digits even with the use of the 2nd and 3rd generation DES devices [9]. It is also noteworthy that ISR developed within DES is especially resistant to further interventional therapeutic approaches and, thus, is often an indication for bypass surgery.

## Vascular Gene Therapy and the Concept of Gene-Eluting Stents (GES)

The use of specialized catheters to deliver and lodge gene vectors in the vessel wall preceded stent-based technologies for vascular gene therapy by 15 years [10]. These initial studies provided important insights in transfectability/transducibility of adventitia, media and intima (neointima) in various experimental species and established multiple potential molecular targets for restenosis prevention [11]. However, the extent of vascular tissue transduction even with sophisticated catheter devices (Dispatch^®^, Infiltrator^®^) remained inadequate for clinical translation of vascular gene therapy mostly due to the short intraluminal retention time of the delivered gene therapeutics [10].

Immobilization of gene vectors on stents embraces the best of both DES and catheter-based methods of vascular gene therapy, by providing the means to overcome the limitations of DES. First, gene therapy can attain a longer lasting therapeutic modification of vascular substrate; second, gene interventions allow for selective inhibition of SMC proliferation and migration while maintaining and even enhancing endothelial re-growth; third, the underlying atherosclerotic process can be addressed; and fourth, a modulation of the biological activity of gene-eluting stents can be achieved with systemically administered low molecular weight compounds.

Furthermore, compared with the scaffold-less vascular gene delivery as accomplished with the use of delivery catheters, stents present an advantageous physical platform for local arterial gene transfer. Indeed, higher arterial concentrations of gene vectors can be achieved with the immobilization on stent surface when compared to non-immobilized vectors following administration of a smaller vector input dose. Superior vascular wall retention is a combined result of physical vector association with a permanently implanted scaffolding device, and a shielding effect of the vector deposited on the adluminal surface of the stent (i.e. at a stent/tissue interface) from the shearing effect of blood flow. As a consequence of protracted vascular residence, immobilization on the stent minimizes the distal spread of gene vector, reducing inadvertent inoculation of non-target tissues. Moreover, stent-based delivery of gene therapeutics is feasible in diseased arterial segments with side branches. Human arteries susceptible to atherosclerosis, with the exception of the common carotid artery, are extensively branched and therefore scaffold-less delivery of fluid phase vector formulations leads to immediate run-off of the vector to the circulation through the side branches. Furthermore, since stent-based gene transfer does not require isolation of the treated vascular segment, procedure-related ischemic complications can be minimized. Finally, stent-based gene delivery provides an optimal spatial configuration to prevent cellular proliferation and migration in the setting of ISR, since cell activation after stent deployment is typically observed in the vicinity of stent struts [12]. Physical association of gene vector with a stent establishes a transgene expression gradient that overlaps with the cell activation gradient, thus approximating vectors to the projected site of the action.

Thus, the use of gene delivery stents for atherosclerotic vascular disease offers the appealing possibility of site specific delivery of gene therapy with long lasting and controllable transgene expression that can prevent in-stent restenosis, and also provide therapy for the underlying vascular disease.

### Minimal requirements for GES function and performance

The design requirements for GES share several characteristics with DES platforms. First, deliverability of both types of devices should not be compromised by the accommodation of a therapeutic agent and its matrix on the stent struts. Second, the matrix associated with DES and GES struts has to withstand the mechanical stretch associated with a change of the stent diameter during deployment. Cracking, peeling and delamination of the matrix during stent implantation should be prevented since these post-deployment defects lead to irregularity in the release rate of a therapeutic agent and can cause distal embolization. Third, the matrix has to be highly biocompatible, causing no more than a minimal thrombogenic and inflammatory response.

There are several important distinctions, however, in the design characteristics of DES and GES. While DES have been devised to be able to provide sustained release and vessel wall delivery of low molecular weight therapeutic agents for as long as 3 months, this may not be necessary for the GES, since transfected/transduced cell populations in the stented artery are able to produce and secrete a transgene product for prolonged period of time. A minimal required duration of stent-associated vector perseverance in its active transduction competent form is not entirely clear. Since the medial and neointimal SMC proliferation starts no earlier than 1 week after stent deployment in human and higher animal models [13], the target cell population is not yet present at the time of vascular injury. This fact argues for at least 2–3 week-long release period of a transduction competent gene vector from the stent, posing additional requirements to GES technology related to extended vector stability *in vivo*. Unlike LMW drugs, which are often stabilized by matrix immobilization, gene vectors (both non-viral and especially virus-based) are vulnerable and lose their activity if not properly protected. Elicitation of immune response to a therapeutic moiety is another specific problem of GES since LMW drugs used in DES are rarely strong immunogenes unless bound to proteins. Therefore, preventing distal vector spread to regional lymph nodes and spleen, as well as physical shielding of the vector from direct contact with pre-formed antibodies and effector T-cells are of paramount importance for sustaining therapeutic levels of transgene expression following GES use. Finally, the 1–2 orders of magnitude size difference between LMW drugs and gene vectors dictates use of different matrices to enable timely release of a therapeutic agent from a stent platform, affecting the predominant mechanism of the agent release (diffusion vs matrix degradation).

## Conceptual Technologies in GES Design

Research concerning gene delivery stents over the past 15 years illustrates a number of principles and mechanisms involved in stent-facilitated delivery of gene therapy vectors to the arterial wall. Several different approaches have been exploited to achieve labile association of gene vectors with stent struts (Table 1).

### Bulk immobilization (polymer coatings)

Initial investigations concerning gene delivery stents utilized polymer coatings on the surface of metallic stents in which gene vectors were dispersed or pseudo-dissolved. These coatings (typically 50–250 μm thick) were deposited on a primed stent surface either by a multiple dip technique or by aerosol deposition of a polymer solution containing a suspension of the vector. After solvent evaporation and polymer precipitation on the stent surface, the gene vector stays contained between the polymer fibers. A subsequent release of vector particles is then controlled by diffusion though the polymer matrix (determined by the relative hydrophilicity/hydrophobicity of the matrix and the pore size), matrix degradation and/or dissolution of matrix in blood and tissue fluid. Depending on the polymer type, these three mechanisms have varying contributions to the overall release kinetics of the vector from the stent coating. A variety of synthetic polymers (PLGA [14], poly(beta-aminoester) [15–18], polyurethane [19,20], PVA [21], poly [phosphorylcholine-lauryl methacrylate] [22–28]), semi-synthetic polymers (dopamine-modified hyaluronic acid [29,30], cationized gelatin [31], styrene-modified gelatin [32], cationized pullulan [33] or naturally occurring macromolecules (collagen [34], denatured collagen [35], gelatin [17,18]) have been employed for the bulk incorporation of genetic material on stent wires.

An obvious advantage of the bulk immobilization is the ability to integrate substantial amounts of the vector in a thick coating. Plasmid DNA quantities as high as 4 mg [20] or Ad doses up to 5×10^10^ particles [34] have been reported. There are several important disadvantages of the bulk immobilization as well. Typically, 80–90% of the vector load is released within the first 24 hours [17,19,21,23,24,33,36], especially in the case of non-viral systems. Part of this effect is due to the “burst release” of a fraction of the vector payload, which is either deposited in the outermost layer of the polymer coating or has been adsorbed onto the polymer layer after going out of solution during the solvent evaporation step. A partial solution for preventing this undesired rapid release of the vector is provided by a super-coating of a vector/polymer composite with additional layers of vector-free polymer [14,17,18]. Another potential method to modulate the release rate of gene therapeutics from bulk polymer coatings uses controlled cross-linking of a polymer as exemplified by Nakayama [32] who developed a photoactivatable coating system consisting of styrene-modified gelatin, a polymerization initiator, carboxylated camphoroquinone and a reporter Ad-LacZ adenovirus vector. Upon irradiation of dipped stents with a broad spectrum halogen lamp, gelatin chains become cross-linked forming a hydrogel that entraps Ad.

Another important disadvantage of the bulk polymer coatings is an inflammatory reaction triggered by native and especially synthetic polymers in the arterial segments placed in contact with the polymers [37]. Thus, unless potent agents, such as the anti-cancer drugs commonly used in drug-eluting stents are utilized, the inflammatory response to the polymer coating negates the therapeutic effect of a transgene. One important exception to the uniformly pro-inflammatory character of polymer coatings is an almost complete lack of inflammatory infiltrates and generally high biocompatibility of poly [phosphorylcholine-lauryl methacrylate] co-polymer utilized in the BiodivYsio stent [23–28] approved by FDA for clinical use in 2000. A hydrophilic poly-phosphorylcholine (PC) moiety of this macromolecule mimics an outer phospholipid layer of cell membranes, thereby reducing platelet activation on the metal surface. Platelet passivation makes this stent a device of choice in patients with absolute contraindications to dual anti-platelet therapy after stenting. A positive surface charge on the BiodivYsio stent imparted by choline residues renders the PC coating useful for electrostatic binding of negatively charged DNA plasmids, as well as Ad and AAV vectors. The latest generation of BiodivYsio stents feature extremely thin coatings (50 nm) precluding significant incorporation of the vector in the bulk of the polymer. Therefore, this system constitutes a paradigm that includes both elements of bulk immobilization and the coatless surface immobilization systems as described below.

### Surface immobilization of gene vectors on coatless metal substrate

Surface immobilization of gene vectors on the surface of endovascular stents represents a particular case of substrate mediated gene delivery, a concept put forward by the work of Shea [38,39] primarily to increase biocompatibility of bioprosthetic scaffolds. The main premise of substrate-mediated gene transfer is to use the tissue-facing surface of an implanted device to append (rather than bulk-incorporate) therapeutic gene vectors in an arranged pattern to facilitate transduction of tissue elements on the interface with the bioprosthesis, thereby improving functional integration of the device.

#### Protein affinity adaptors for vector immobilization

Studies by our group further developed the idea of substrate mediated delivery for use in conjunction with endovascular stents. Driven by the established data on detrimental pro-inflammatory and pro-restenotic effects of stent coatings we designed and implemented a chemical strategy for reversible attachment of both viral and non-viral gene vectors that does not rely on surface coating. Our method of vector immobilization on non-coated metal surface is based on surface modification with poly(allylamine)-bisphoshonate (PAB) compounds bridging the metal surface and gene vectors [40]. Bare metal gene delivery using PAB involves first forming a polymer monolayer on the metal surface of the stent that is established due to the formation of strong coordination bonds between pendant bisphosphonic groups of the polymer and Fe, Ni and Cr atoms and their oxides on the steel surface. This ultra-thin (less than 5 nm) permanently attached polymer film is rapidly formed upon exposure of metal samples to an aqueous PAB solution. The molecular monolayer of PAB can be further covalently modified in order to permit a covalent attachment of vector binding agents using well established amine conjugation chemical strategies targeting multiple primary amines of the allylamine backbone [40]. High affinity vector binding proteins such as anti-Ad knob [40], anti–DNA [41] antibodies or a recombinant D1 domain of Coxsackie-Adenovirus Receptor [40] can be used as binding adaptors enabling high-affinity binding of gene vectors to stents. Stent loading with gene vectors is then achieved by simple immersion of a stent in a vector suspension. We further demonstrated that agents with different binding affinities can be exploited to adjust a vector capacity of the gene-eluting stent and to modulate the release rate of gene therapeutics both *in vitro* and *in vivo* [40]. However, limited control over the vector release kinetics remains a disadvantage of the protein affinity binding immobilization technology. Although we have demonstrated the vector presence at the interface between stent struts and the arterial wall 24 hours post-deployment and associated reporter (GFP) activity in all 3 arterial layers 7 days post-stenting [40], the duration of the vector binding to a stent *in vivo* may be far from optimal. It is challenging to prolong vector association with the stent surface using this technique, since the vector association with stents is determined by the antigen/antibody or receptor/ligand affinity, local pH, and the protein content of blood and tissue fluid; the factors that cannot be changed deliberately. Furthermore, the protein affinity binding technology cannot be immediately adapted to any given vector, since it implies availability of a vector-binding molecule with Kd in the range of 10-8–10-9 M. Moreover, vector-binding strategies based on protein affinity interactions are difficult to adapt to manufacturing scale because of protein stability and species specificity issues.

#### Viral vector tethering to bare metal stents via hydrolyzable cross-linkers

**Figure 1:**
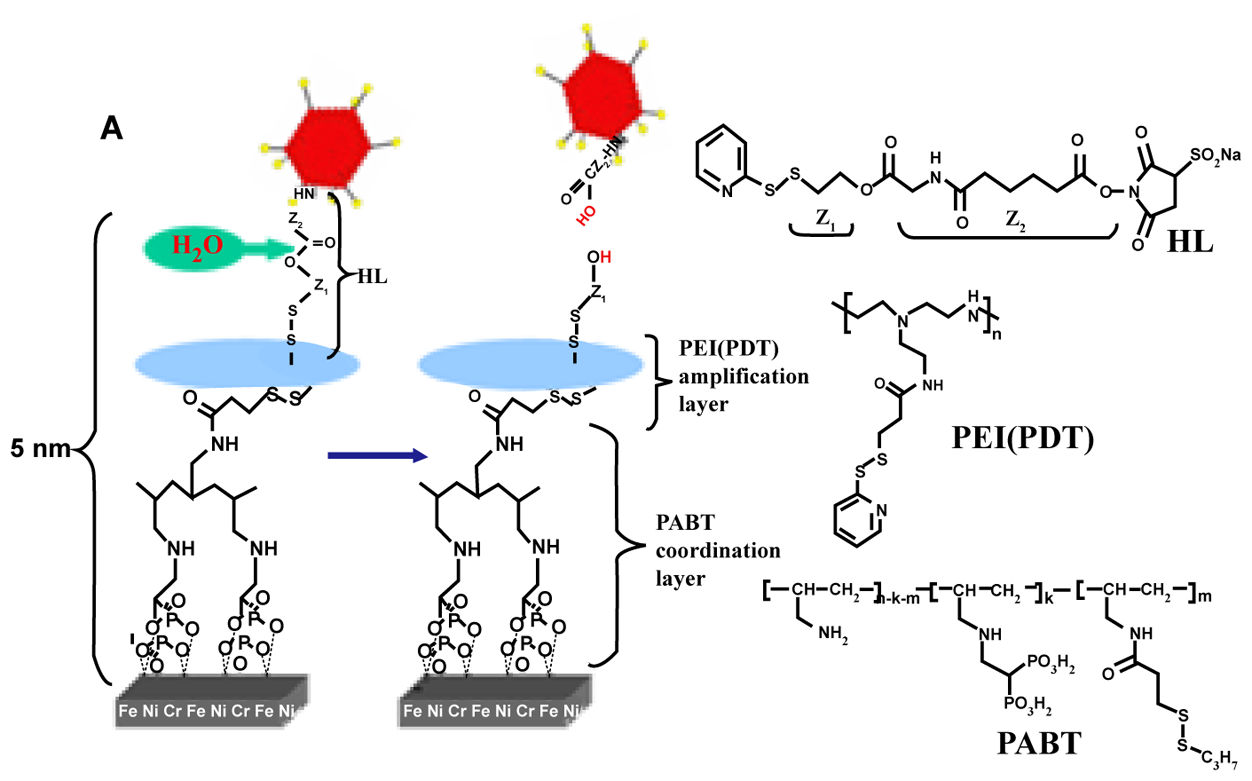
A scheme illustrating specific chemical interactions to enable adenovirus binding to a stent surface. Ad vectors were modified by reacting lysine residues of capsid proteins with a bifunctional amine/thiol-reactive Hydrolyzable Cross-Linker (HL) possessing a hydrolyzable ester bond separating fragment Z1 and Z2. Stainless steel stents were consecutively exposed to a solution of Polyallylamine Bisphosphonate Comprising Latent Thiol Groups (PABT) and a reducing agent, TCEP, to activate thiol groups on the surface. To expand the amount of available thiol functions, a subsequent treatment with polyethyleneimine modified with pyridyldithio groups, PEI (PDT), and DTT was used. Finally, HL-modified Ad vectors were reacted with thiolated stent surfaces to achieve covalent tethering of Ad. The subsequent release of covalently immobilized Ad is driven by hydrolysis of the ester bond in the cross-linker’s backbone. (Adapted from [42] with permission).

Motivated by the limitations of affinity immobilization we developed an alternative methodology for the reversible binding of recombinant replication-defective adenoviruses to metal surfaces that completely avoids using protein adaptors. This method is based on hydrolyzable cross-linker (HC) molecules that directly append vectors to PAB-activated steel (Figure 1). The subsequent release of the vectors is governed by the kinetics of cross-linker hydrolysis (Figure 1) and can be modulated by the usage of HC with variable hydrolysis rates. A new conjugation strategy necessitated a change of PAB chemical design with the introduction of latent thiol groups in the side chains of the polymer molecule. This novel compound, PABT, was successfully synthesized and characterized by our group [42]. The devised linking strategy by itself was sufficient to achieve significant binding of thiol-reactive Ad particles to the surface of model steel samples. However, we choose to expand the amount of available thiol group on the metal surface using additional exposure of thiol-activated metal samples to an aqueous solution of pyridyldithio (PDT)-engrafted polyethyleneimine, PEI (PDT) followed by reduction of PDT to thiols with dithiothreitol. We demonstrated that the “amplification” protocol resulted in a more effective Ad tethering when compared with the basic “no-amplification” protocol [42].

Synthetic, biodegradable HC are particularly promising tools for achieving a site-specific tunable release of gene therapy vectors since they can be synthesized to have a broad range of hydrolysis rates and thus the vector release rate from the surface of a stent can programmed per the formulation parameters [43].

### Magnetic stent targeting

Both bulk immobilization and surface tethering approaches make use of stent-based gene delivery systems that are assembled prior to stent implantation in the artery. Introduction of a stent into the vasculature through the hemostatic valve of a vascular sheath and routing it to a deployment site through the atherosclerotic and often calcified arterial conduit always involves some physical damage to the stent coating and vector depot. As an alternative approach, stent loading can be accomplished *in situ* after the stent has already been implanted. Since the deployed stent is freely accessible to blood flow, its surface can be actively targeted with therapeutic agents delivered to systemic or regional circulation, provided the targeting forces are strong enough to capture and retain a therapeutic agent on the stent surface.

**Figure 2:**
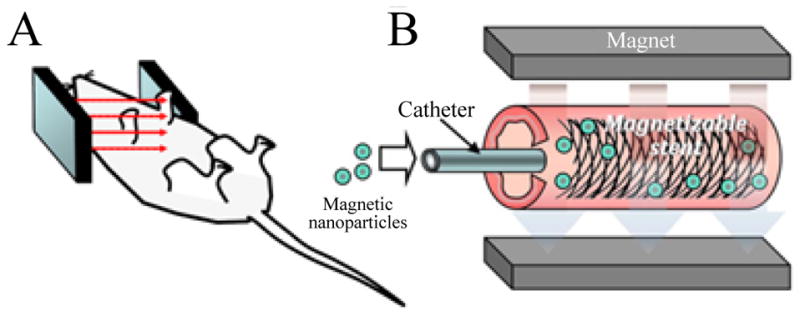
A targeted delivery of MNP co-formulated with Ad-Luc vectors to a deployed stent mediated by the uniform field induced magnetization effect. The uniform field generated by paired electromagnets (A) both induces high gradients on the stent and magnetizes Ad-loaded MNP, thus creating a magnetic force driving MNP to the stent struts and adjacent arterial tissue (B). (Adapted from [45] with permission).

This concept of *in situ* stent loading with gene vectors was recently implemented by our group using a magnetic targeting paradigm [44]. Stents made of magnetic alloys, when placed in a uniform magnetic field, such as that created within an MRI coil or induced by paired electromagnets generate strong highly localized magnetic forces due to steep field gradients between stent struts [44–47]. These magnetic forces enable the targeted capture of systemically or locally administered gene vectors formulated in MNP [44–47] (Figure 2). The same external magnetic source can be used to magnetize both stents and MNP. In our study [44], after implanting stents made of magnetically responsive 304-grade stainless steel in the common carotid arteries of rats, suspension of MNP incorporating 2.5×109 Ad-Luc was slowly injected through the catheter into the aortic arch. The delivered vector was then transported en masse with antegrade blood flow to carotid circulation across the stented segment. During and immediately after MNP/Ad-Luc delivery a uniform magnetic field of 0.1 Tesla was established across the neck area of the rats. While no direct quantification of the amount of Ad vector attached to stent struts following magnetic targeting was pursued, the vector uptake was significant enough to cause a localized transduction of stented carotid segment, greatly exceeding that achievable with free Ad, as determined by bioluminescence imaging [44].

#### Magnetic reloading of depleted GES

Prolonged arterial expression of a transgene with an established anti-restenotic activity following a single intervention is the ultimate goal of stent-based vascular gene therapy. Despite ongoing research in the field for the past decade, it is still too early to conclude whether this goal is realistic. Indeed, even with protracted release kinetics of gene vectors from stents, such as one achieved with hydrolyzable cross-linker immobilization, the duration of the transgene expression and hence the durability of the therapeutic effect is a concern. In most mammalian tissues, after reaching peak level, transgene expression tends to decrease over time due to both promoter attenuation and mounting immune response to the vector and the transgene product. Previous reports [48,49] have documented that suppressed neointimal growth can recur if the inhibiting modality is withdrawn early. The vulnerability period for restenosis recurrence following premature treatment withdrawal in human arteries is unknown, but appears to be longer for peripheral compared to coronary vasculature. Therefore, the potential to rejuvenate therapeutic transgene expression by reloading stents with additional payloads of the same or other gene vectors is of paramount importance in designing translatable vascular gene therapy approaches to prevent restenosis, especially in peripheral vasculature. Uniform field high-gradient magnetic targeting provides a potential way of addressing reloading of depleted GES at delayed time points. In order to reload a stent *in situ*, a gene vector needs to be physically partitioned at or near the depot for sufficient time to saturate binding sites in the depot matrix with the re-delivered vectors. Magnetic field mediated targeting provides a unique physical opportunity to achieve this task.

## Reporter Studies and Pharmacokinetics of Transgene Expression

Reporter studies are instrumental for optimizing stent-based gene delivery systems in regards to transgene expression strength and durability. GFP [14,16,20,34–36,40,42,50,51], β-galactosidase [20,21,23,26,27,32] and luciferase [20,36,40,42,43] have been extensively employed to map temporal and spatial expression patterns of stent-delivered transgenes. It is noteworthy that the information provided by different reporter systems is not redundant. For example, while luciferase is typically more sensitive than the other two reporter systems, it does not readily provide a tissue level resolution. Use of GFP and β-galactosidase, on the other hand, is compatible with direct histological examination of transduced tissue, providing valuable information about transduced cell populations and their distribution in distinct compartments of the vessel wall. Native GFP fluorescence is labile and extremely sensitive to tissue fixation methodology. Moreover, GFP emits light in the same part of spectrum as elastin, making unambiguous detection of GFP positive cells difficult, especially in the injured tissue, where elastin is partially disintegrated. A recent advent of high quality commercial anti-GFP antibodies makes this task easier by streamlining immunofluorescence and immunohistochemical approaches for the identification of transduced cells.

A direct comparison of the extent and duration of reporter arterial expression among the published studies is challenging because of dissimilarities between animal models, reporter systems and reporter detection methods employed by different groups. Nevertheless, several common traits of arterial tissue transduction from a stent platform are preserved throughout most of these studies regardless of the specific experimental setup. First, gene transfer is highly site-specific, leading to more effective transduction of cells underlying stent struts compared to cells located between the stent wires [20,21,26,27,40,42]. Second, despite neointimal growth not occurring until 4–14 days after a stent deployment, a nascent neointima is more effectively transduced than media and adventitia [20,26,36,40,42]. Third, duration of gene expression may exceed one month following implantation of GES [26,27]. Fourth, no massive distal spread of a transgene to liver, spleen and lungs is apparent [14,20,34,40,42]. Fifth, different vector systems may have predilection for transducing specific cell populations in the arterial wall [20,26].

## Disease-related Targets and Therapeutic Transgenes

In-stent restenosis is a multifactorial disease [52,53]. The stenting related factors with proven pathogenic bearing for ISR include endothelial denudation, direct trauma to medial SMC resulting in altered SMC apoptosis, proliferation and ECM synthesis programs, parietal thrombus formation, unresolved inflammation and foreign body reaction to the stent and its coating. Accordingly, the number of molecular targets relevant to inhibition or stimulation of signaling pathways altered during ISR development is extremely high [52,53]. Comprehensive reviews [11,54,55] focused on gene therapy of restenosis and related vasculoproliferative conditions list more than 150 different genes implicated in regulation of cell proliferation, migration, ECM turnover, thrombosis and inflammation that were targeted with gene therapy interventions to mitigate restenosis. Only a small fraction of these therapeutic gene constructs was investigated in conjunction with GES.

### GES targeting re-endothelialization

Since the seminal study by Asahara et al. [56] describing inhibition of restenosis in a rat model with locally administered vascular endothelial growth factor (VEGF) was published in 1995, strategies aimed at attenuating restenosis via enhanced re-endothelialization have come into focus. In line with this concept, Walter et al. [28] have used poly[phosphorylcholine-lauryl methacrylate]-coated stents to electrostatically adsorb 200 μg plasmid DNA encoding human VEGF-2. When deployed in Fogarty balloon-denuded external iliac arteries of hypercholesterolemic rabbits, VEGF-2 plasmid-eluting stents enhanced endothelial recovery in the stented arteries in comparison with the animals receiving control stents and resulted in a 60% reduction of cross-sectional arterial narrowing of the stented region 3 months after the procedure [28].

### GES targeting SMC proliferation, migration and ECM remodeling

Serine/threonine kinase, Akt1, also known as protein kinase B (PKB) is an important signaling hub integrating input from several pro-proliferative pathways, in particular from PI3K [57]. Akt1 activation during neointimal tissue maturation [58] as well as the anti-restenotic effectiveness of Akt1 inhibition [59] are well established. Recently

Che et al. [29] applied coordination chemistry to achieve stable attachment of dopamine-conjugated hyaluronic acid (HA) to stents via bonds formed between dopamine residues and metal ions on the stent surface. The HA-coated stents were further modified with polyplexes made of siRNA to Akt1 and disulfide cross-linked PEI. A sustained release of siRNA from the stent surface was observed *in vitro* for at least 72 hours. In *in vivo* study, an undisclosed amount of Akt1 siRNA polyplexes was deposited on HA-coated stents. Akt1 siRNA stents implanted in rabbit iliac arteries decreased the extent of ISR by 50% at 4 weeks after deployment compared to BMS [29].

Platelet-derived growth factor (PDGF) existing in two homodimeric forms (PDGF-AA, PDGF-BB) and as a heterdimer (PDGF-AB) is a well established SMC mitogen [60] and pro-migratory chemoattractant [61]. All three isoforms were shown to promote SMC accumulation *in vitro* and *in vivo*, while demonstrating relatively low activity toward endothelial cells [62]. Therefore, LMW inhibitors of PDGF receptor tyrosine kinase activity [63], as well as gene therapy modalities of inhibiting the PDGF-triggered signaling cascades [64,65] are appealing directions to selectively inhibit SMC growth without affecting endothelial cell regeneration. To this end, Li et al. [66] synthesized phosphorothioate antisense oligodeoxynucleotides (ODN) to the 15 base-long conserved coding sequence of PDGF-A gene and complexed them with PEI to make a polyplex formulation that was absorbed on hydrogel coated stents. When implanted in the coronary arteries of healthy young pigs, the anti-sense-eluting stents resulted in a 50% reduction of neointimal thickening in comparison with non-sense ODN-eluting and plain hydrogel stents [66]. Importantly, the endothelial lining was complete 28 days after stenting in the arterial sections from the animals treated with the antisense PDGF-A–eluting stents, while the endothelialization was patchy at the 28 days time point in the arteries of animals receiving commercial sirolimus-eluting stents [66]. Likewise, complete endothelialization of stented pig coronaries and a 40% reduction of restenosis were observed with a six-ring phosphorothioate morpholino backbone anti-sense construct eluted from a PC-coated stent and targeting expression of a cell cycle check point transcription factor c-myc [67].

In-stent neointima is primarily formed by the progeny of medial SMC and adventitial myofibroblasts that populate the intimal space of an injured artery. Matrix remodeling triggered by vascular trauma makes possible unhindered migration of these cell populations along the gradients of specific chemoattractants [68]. Thus, stabilization of ECM was proposed for limiting SMC migration toward (neo) intima. In accordance with this idea, Johnson and colleagues [23] bulk-immobilized a suspension of Ad vector (4×108 pfu) encoding synthesis of the tissue inhibitor of metalloprotease-3 (TIMP3) on PC-coated stainless steel stents. At 28 days after stent implantation in pig carotid arteries neointimal volume was reduced by 40% and 50% in the animals treated with Ad-TIMP3-decorated stents in comparison with pigs receiving BMS or Ad-LacZ immobilized stents, respectively [23].

### GES targeting inflammatory cell recruitment

Acute inflammatory response in the form of platelet and fibrin deposition and massive neutrophil recruitment to the site of vascular trauma via P-selectin and CD11b/CD18 engagement is triggered immediately upon stent deployment [52,69]. Days later, an escalating production of IL-6, IL-8 and MCP-1 by the injured SMC and ingressing neutrophils attracts monocytes that transmigrate into the tissue becoming resident macrophages thus denoting a stage of chronic inflammation. Macrophages are the key cell type bridging inflammation to neointimal formation, since macrophage-produced and secreted chemokines (TNF, IL-1β) and growth factors (PDGF, FGF-2, IGF) stimulate luminal migration and proliferation of medial SMC and adventitial myofibroblasts that collectively form neointima [52,69,70]. Therefore, effectively curbing the inflammatory response represents a well-recognized anti-restenotic strategy [71,72].

In keeping with this therapeutic approach, Egashira and co-workers [21] evaluated stents formulated with plasmid DNA encoding dominant negative variant of MCP1. The stents deployed in the iliac arteries of hypercholesterolemic rabbits and cynomolgus monkeys resulted in approximately a 20% reduction of macrophage infiltration at the stent implantation site and a 20–30% restenosis attenuation in comparison with BMS [21].

NFkB is a master transcription factor involved in the regulation of multiple inflammatory mediators. Ohtani et al. [19] used NFkB decoy ODN (500 μg/stent) incorporated in a polyurethane coating. Stents deployed in femoral arteries of hypercholesterolemic rabbits demonstrated approximately a 30% decrease of neointimal thickening in comparison with BMS and polyurethane coated stents at 4 weeks post-stenting and a significant decrease of inflammatory marker expression at 3 and 10 days post-implantation [19].

### GES targeting thrombus formation in stented arteries

Platelets are the first cellular elements to contact the surface of newly deployed stents. Since both human patients and experimental animals undergoing stent placement are pretreated with anti-platelet and anti-coagulant drugs, acute blockage of a treated artery is seldom an issue. However, a non-occluding parietal thrombus is routinely formed at the angioplasty/stenting site. If the parietal thrombus persists, it gets populated with the migrating SMC, thus facilitating fast neointimal growth.

In an attempt to minimize platelet activation at the site of stent implantation, Takemoto et al. [31] formulated gene-eluting stents incorporating plasmid DNA encoding E-NTPDase, a crucial regulator of platelet thrombus formation. This molecule is a membrane bound enzyme that locally hydrolyses ATP, thus opposing platelet aggregation. The plasmid DNA (78 ± 5 μg) was absorbed into cationized gelatin hydrogel coating on the stent struts. Implantation of E-NTPDase-eluting stents in a rabbit model of repeated femoral injury, which is characterized by a high rate of thrombus formation completely, prevented the occurrence of intravascular thrombi, while 25% and 50% of femoral arteries treated with control βGal plasmid-eluting stents had compromised patency at 3 and 7 days post-stenting. Moreover, since thrombus serves as a nidus for SMC colonization, thus enhancing neointimal formation, the extent of early restenosis (at day 7) was significantly reduced in the vessels treated with E-NTPDase stents [31].

### Stents targeting nitric oxide production

Nitric oxide (NO), a by-product of enzymatic conversion of arginine to citrulline has recently emerged as a key signaling molecule in vascular homeostasis [73]. Increased NO production and availability were shown to prevent platelet aggregation [74], inhibit SMC proliferation [75,76], and migration [77], promote endothelial growth [78], and decrease expression of cell adhesion molecules by endothelium, inhibiting ingress of activated leukocytes into the arterial intima [79]. Additionally NO promotes viability of endothelial progenitor cells and enhances their capacity to reconstitute damaged endothelium [80]. The pleiotropic nature of its beneficial actions makes NO-related interventions a plausible strategy for the prevention of ISR.

Both endothelial [81,82] and inducible [83,84] forms of NO synthase (eNOS and iNOS, respectively) were investigated as genes with potential anti-restenotic activity in animal models of arterial injury/local delivery generally showing strong inhibiting effects on neointimal formation. Driven by the wide scope of anti-restenotic mechanisms bestowed by NO, we [40,42] and others [17,25,26,51] have pursued the idea of the stent-based delivery of NOS-encoding vectors.

The studies by Sharif and others [25] employed PC coated stents configured with 5×109 pfu of Ad-eNOS to address the hypothesis that enhanced reendothelialization and reduced restenosis can be achieved in a rabbit model with GES-induced eNOS overexpression. The authors demonstrated significantly enhanced restoration of the endothelial layer and reduced neointimal thickness in both normo- and hypercholesterolemic rabbits treated with Ad-eNOS stents in comparison with animals that received Ad-βGal-eluting stents or blank PC-coated stents. In an attempt to formulate a gene-eluting stent devoid of immunogenic and pro-inflammatory adenoviral proteins, thus facilitating clinical translation of stent-based eNOS overexpression, the same group recently reported [26] on a Lipofectin-based lipoplex formulation containing human eNOS plasmid. Ten μg of the lipoplex formulation were uniformly deposited on PC-coated stents. The non-viral gene-eluting stents deployed in iliac arteries of hypercholesterolemic rabbits achieved levels of transgene expression comparable to Ad-containing GES. Moreover re-endothelialization at 28 days was similarly enhanced by eNOS lipoplex-eluting stents. However, no significant reduction in restenosis indices was observed in this study. The authors explain this discrepancy by the fact that liposomal formulation primarily targets the macrophage population in the diseased artery and not SMC, thus missing the vital cell type affecting restenosis.

In contrast, using a similar rabbit model for therapeutic assessment of eNOS-lipoplex-eluting stents Brito [17] demonstrated a 30% reduction of restenosis accompanied by a 50% decrease of proliferation in the eNOS lipoplex-treated rabbit arteries in comparison with an empty vector control. The difference in the vector dose (10 μg vs 25 μg) and the delivery system used (adsorption of lipoplexes on a PC-coated stent vs stent coating with lipoplexes dispersed in a gelatin/mannitol formulation with additional lipoplex-free gelatin/PLGA supercoating) between the former and the latter studies may account for the different outcomes, highlighting the importance of proper pharmaceutical adjustments for revealing full therapeutic potential of GES.

In our studies, Ad-iNOS delivered in a rat model from PAB- or PABT-modified bare metal stents using affinity adaptors [40] or hydrolyzable cross-linkers [42] for vector tethering, respectively, resulted in a 50%–60% reduction of neointimal thickening in comparison with the BMS-implanted arteries. These effects were achieved using 109–1010 Ad-iNOS particles (107–108 pfu) appended to a stent. Our recent studies assessing NO synthesis and ROS production in cultured SMC and endothelium transduced with Ad-iNOS tethered to stainless steel disks have determined that the super-physiological levels of iNOS expression driven by the immobilized vector can lead to NOS decoupling due to deficiency of arginine and tetrahydrobiopterin [85]. Decoupled NOS is unable to generate NO, producing superoxide instead [86]. If not effectively removed by superoxide dismutases, superoxide triggers a sequence of redox reactions that result in the formation of multiple additional ROS, which can cause extensive tissue damage [86]. Therefore, pharmacological supplementation with arginine and tetrahydrobiopterin may be required to modulate the function of NOS overexpressed in the arterial wall using stent based delivery of a respective vector [85].

Which of three known isoforms of NOS is the best choice for the use in conjunction with GES is not immediately obvious. A study by Cooney [82] compared the antirestenotic effectiveness of Ad-iNOS and Ad-eNOS in a model of intraluminal dwell delivery of the vectors to temporarily isolated segment of balloon-injured rabbit carotid arteries. While a 20-min incubation of both vectors (109 pfu) resulted in an identical extent of neointimal inhibition in comparison with Ad-βGal-treated counterparts, this was allegedly achieved by dissimilar mechanisms, since eNOS overexpression promoted and iNOS overexpression inhibited endothelial regeneration in the treated arterial segments [82]. Additional in-depth studies are required to elucidate the actual mechanisms involved in the antirestenotic effects of different NOS isoforms and their utility in the setting of GES-induced overexpression.

## Conclusions

Gene-eluting stents represent an interesting alternative to drug-eluting stents in clinical circumstances where current DES formulations often fail to provide satisfactory results. Research related to GES has now come to a phase when the perspectives of clinical translation should always be taken into consideration. However, further work related to optimization of all three main design components of GES: a therapeutic transgene, a vector and a delivery system has to be done before stent based vascular gene therapy comes to clinical fruition.

## Figures and Tables

**Table 1:** Comparison of gene vector immobilization methods on stent surfaces.

Immobilization method	Advantages	Disadvantages
*Bulk coating*	High loading of gene vectors, ease of scale-up	Inadequate release kinetics, polymer-induced inflammation
*Surface tethering*	Controllable release rate, no inflammation	Low loading of gene vectors
*In-situ stent loading using magnetic targeting*	No vector loss on route to delivery site, possibility of repeated loading	A limited choice of stent materials, MNP safety issues

## Abbreviations

Ad: Adenovirus
AAV: Adeno-Associated Virus
BMS: Bare Metal Stents
DES: Drug-Eluting Stents
ECM: Extracellular Matrix
E-NTPDase: Ectonucleoside Triphosphate Diphosphohydrolase
GFP: Green Fluorescent Protein
FGF-2: Fibroblasts Growth Factor-2
HC: Hydrolyzable Cross-Linker
IGF: Insulin-Like Growth Factor
IL-1β: Interleukin-1β
IL-6: Interleukin-6
IL-8: Interleukin-8
ISR: In-stent Restenosis
LMW: Low Molecular Weight
LST: Late Stent Thrombosis
MCP-1: Monocyte Chemoattractant Protein-1
MNP: Magnetic Nanoparticles
NFκB: Nuclear factor κB
NO: Nitric Oxide
NOS: Nitric Oxide Synthase
ODN: Oligodeoxynucleotides
PAB: Polyallylamine Bisphosphonate
PABT: Polyallylamine Bisphosphonate with Latent Thiol Groups
PC: Poly-Phosphorylcholine
PCI: Percutaneous Coronary Interventions
PDGF: Platelet-Derived Growth Factor
PEI (PDT): Polyethyleneimine Modified with Pyridyldithio Groups
PFU: Particle Forming Units
PLGA: Poly [lactic-co-glycolic acid])
PVA: Poly Vinyl Alcohol
ROS: Reactive Oxygen Species
SMC: Smooth Muscle Cells
TIMP-3: Tissue Inhibitor of Metalloproteinases-3
TNF: Tumor Necrosis Factor
VEGF: Vascular Endothelial Growth Factor
